# Estimated epizoochory seed dispersal distances by grazing yak across seasons in an alpine meadow

**DOI:** 10.3389/fpls.2025.1569043

**Published:** 2025-06-12

**Authors:** Shulin Wang, Fujiang Hou

**Affiliations:** ^1^ State Key Laboratory of Herbage Improvement and Grassland Agro-ecosystems; Key Laboratory of Grassland Livestock Industry Innovation, Ministry of Agriculture and Rural Affairs; Engineering Technology Research Center for Ecological Restoration and Utilization of Degraded Grassland in Northwest China, National Forestry and Grassland Administration; College of Pastoral Agriculture Science and Technology, Lanzhou University, Lanzhou, China; ^2^ Yunnan Key Laboratory of Plateau Wetland Conservation, Restoration and Ecological Services, Southwest Forestry University, Kunming, China; ^3^ National Plateau Wetlands Research Center, College of Wetlands, Southwest Forestry University, Kunming, China

**Keywords:** adhesive structure, behavioral observations, grazing behavior, seed detachment, seed retention time, yak

## Abstract

**Introduction:**

Epizoochorous dispersal of grassland plants by large herbivores is an important way by which grassland plants achieve population expansion over long distances. However, little is known about the maximum distance that seeds can be dispersed by domestic animals under seasonal grazing, which is the most common type of grassland management worldwide, especially in alpine regions.

**Methods:**

To this end, we estimated the distance over which epizoochory dispersal occurs via yaks (*Poephagus grunniens*) for seven common plant species seeds in an alpine meadow under seasonal grazing using a simulated yak-fur seed adhesion test combined with observations of grazing behavior.

**Results:**

The results showed that, as yak primary (e.g., walking time) and secondary (e.g., foraging rate) behavioral patterns differed significantly across seasons (*P*< 0.05), the epizoochory dispersal distances of plant seeds also had clear seasonal dynamics, manifesting as spring > summer > autumn > winter, and that the length of seed adhesive structures correlated positively with the retention rate as well as retention time on yak fur. The relatively slow loss of diaspores observed in this study mainly shows that moving yak from one seasonal pasture to the following allows the dispersal of diaspores between two successive pastures. The dispersal scale was even wider (maximum dispersal distance of ~35 km) for seeds with special appendages (i.e., mucilage, sticking to the fur due to mucilage presence).

**Discussion:**

Our results highlight that yaks are substantial seed dispersal vectors for alpine meadow plants and that seasonal grazing is a suitable management method for coping with habitat fragmentation as well as plant diversity conservation in alpine areas from the perspective of seed dispersal.

## 1 Introduction

Seed dispersal is a key process in the plant life cycle. The dissemination of seeds away from the parent plant greatly reduces competition among the resulting offspring and thus is conducive to the survival and reproduction of the entire population as well as the increased biodiversity and spatial distribution of the species. Animal dispersal (i.e., zoochory) is an important mechanism for spreading plant seeds, with half of plant species worldwide requiring animals to spread their diaspores (Fricke et al., 2022). Animals can spread plant seeds both through their digestive tract (i.e., endozoochory) and externally when attached to their bodies (i.e., epizoochory) (Couvreur et al., 2005; Albert et al., 2015). As compared to endozoochory dispersal, epizoochory is a process that relies on physically carrying the seeds, such that seeds that adhere to the animal’s body surface are not subjected to a series of destructive effects such as mechanical mastication (ruminating), exposure to chemical ruminal and intestinal digestive fluids, and fecal erosion (Manzano and Malo, 2006). Therefore, for less robust, resistant, or protected plant seeds, epizoochory may be more advantageous than endozoochory.

In grassland ecosystems, epizoochory by large herbivores is an important mode of seed dispersal for grassland plants, and seeds that adhere to the fur of ungulates (e.g., domestic livestock) constitute what is referred to as the fur seed bank (Wang et al., 2021). It is generally believed that seeds with adhesive structures (e.g., awns, stiff hairs, bristles, hooks, mucilage, etc.) are necessary for epizoochory dispersal because they are more likely to adhere to animal fur (Couvreur et al., 2004; Anders et al., 2009; Albert et al., 2015; but see Green et al., 2022). Moreover, seeds with specialized adhesive structures can be retained on animal fur for a relatively long period, thus providing the possibility of long-distance dispersal (Manzano and Malo, 2006). It is the range and behavior of the animal, as the dispersal vector, that determine the fate of seeds adhering to its fur, i.e., the final distance of seed dispersal and the likelihood of reaching a suitable habitat for germination (Albert et al., 2015).

As the broadest terrestrial ecosystem on the planet, grasslands cover ~40% of the land surface area (Maestre et al., 2022). Grassland ecosystems are generally characterized by distinct seasonal patterns, such as the warm and cold seasons of the typical steppe in northern China (Shi et al., 2022), the dry and rainy seasons of the savanna in Africa (Sitters et al., 2014; Wells et al., 2023) and the rainy winters and dry summers of Mediterranean grasslands (i.e., rain heat synchronization) (Metz et al., 2010). Annual variations in temperature, precipitation and photoperiod lead to alternating growing and non-growing seasons (Fischer et al., 2023). Plants respond to these seasonal changes in abiotic conditions by adjusting the timing of their life cycle (i.e., phenology) (Moulin et al., 2021; Wang et al., 2024), which results in obvious seasonal dynamics of herbage production and supply that manifest as a surplus during the growing season and a deficit during the non-growing season (Sun et al., 2015). Therefore, seasonal grazing (e.g., transhumance, which involves seasonal drives of animals for hundreds of kilometers in search of productive pastures, Manzano and Malo, 2006) is the most common management mode for natural grasslands worldwide (Manzano et al., 2005; Talore et al., 2015; Tian et al., 2021; Shaheen et al., 2025).

The dispersal distance of plant seeds by epizoochory of herbivores is a central issue in the field of seed ecology. As compared to other dispersal mechanisms, zoochorous dispersal occurs across greater distances (Manzano and Malo, 2006) and thus has the potential to open up new habitats for offspring. Because of the different behavioral traits and activity patterns of large ungulates under seasonal grazing (Liu et al., 2019), epizoochorous dispersal distances of herbage seeds are also highly variable. Many seeds are capable of adhering to the fur of yaks—the most widespread livestock species on the Qinghai-Tibetan Plateau region. For example, in one study the density of the fur seed bank of yaks grazing in the warm season (late August) and cold season (mid-October) in this region was 585.27 and 218.18 seeds/m^2^, respectively (Wang et al., 2021). To our knowledge, however, no studies have been reported on the epizoochorous distances of plant seeds transported by yaks as well as other livestock species in alpine regions under seasonal grazing.

To this end, we used a simulated yak-fur seed-adhesion test combined with grazing behavior observation data in an alpine meadow on the Qinghai-Tibetan Plateau. This study aims to answer the following questions: (1) how does the length of seed appendage structures relate to the retention rate and retention time on yak fur, and (2) what is the estimated epizoochory dispersal distance of seeds on yaks under seasonal grazing? This study consisted of two separate experiments. First, a simulated yak-fur seed-adhesion test was carried out to determine the retention rate as well as retention time on yak fur. Second, yak grazing behavior across seasons was observed and used to estimate epizoochorous seed dispersal distances. This study may provide a reference for assessing the epizoochorous dispersal distance of plant seeds by large herbivores across seasonal pastures.

## 2 Materials and methods

### 2.1 Study area

This study was carried out in Maqu County, Gannan Tibetan Autonomous Prefecture, Gansu Province, China (101°50′ E, 33°40′ N, 3550 m a.s.l.), which is situated on the northeastern part of the Qinghai-Tibetan Plateau. The area has a plateau-type climate with cold temperatures and relatively high humidity and only two seasons, namely the warm season (early May to late November) and cold season (early December to late April). For the convenience of distinguishing between different grazing periods for this study, we divided the warm season into a summer (early June–late August) and autumn (early September–late November) and the cold season into a winter (early December–late February of the next year) and spring (early March–late May). The vegetation type is alpine meadow, and the specific soil type is alpine-meadow soil (see **Supplementary Material Appendix** for details). During the study period (early January to late October, 2022), the average annual temperature was 3.0°C and the average annual precipitation was 639.3 mm (**Supplementary Figure S1**).

### 2.2 Seed adhesion simulation test

Yaks are semi-feral animals (Liu et al., 2023), with natural dispositions that can vary from timid to aggressive; non-domesticated yaks cannot be easily approached. The yaks observed in this study had long, rough, undulated hairs with an average length of ~5 cm and coarse hairs with an average length >20 cm. When yaks walk, rest/lie down and wallow in their grassland environment, diaspores (i.e., dispersal units, hereafter referred to as seeds) will adhere to their fur, but those seeds cannot be easily assessed. This is why we used simulation experiments in the present study. Fur-mimicking adhesion tests are regularly used in studies related to zoochory seed dispersal, especially for wildlife (Kiviniemi, 1996; Couvreur et al., 2004; Anders et al., 2009).

Six common plant species seeds with obvious adhesive structures—*Elymus nutans*, *Stipa purpurea*, *Stipa capillacea*, *Anemone rivularis*, *Anemone coelestina*, and *Rumex patientia* (**Supplementary Table S1**)—were selected to analyze seed retention rates as well as retention times on yak fur (**Supplementary Figure S2**). In addition, some seeds are able to secrete mucilage when exposed to water (i.e., moisture), which functions as a special dispersal-assisting structure, and such seeds could adhere to animal fur, leading to long-distance dispersal. Considering the rainy climatic characteristics of the study area, we therefore analyzed an additional species of seed, the mucilage-producing seeds of *Salvia roborowskii* (**Supplementary Figure S3**). Importantly, the seeds from the selected seven plant species are all characterized by persistence, with mature seeds being retained on the reproductive branches of these plant species for several months (i.e., they constitute the canopy seed bank), and the greater height of these plant stems ensures that the seeds can easily adhere to the yak belly or flank fur, even when the animal is walking/standing (**Supplementary Figure S4**).

The length of the adhesive structures of each seed species was measured with an electronic vernier caliper (**Supplementary Figure S2**), with 12 seeds measured for each species. Then, a full yak skin was purchased from the market and was cleaned to ensure that no existing seeds were present. The cleaned yak skin was cut into 15-cm × 15-cm square pieces, each of which was fixed on a 20-cm × 20-cm wooden board with nails, and this board was then affixed to a 50-cm-long wooden strip, which served as a stake. Yaks have different fur types across their bodies, and all skin pieces cut from the flank section as this section has the largest area. The signboards were staked in a plot of natural grassland, with 2 m between each signboard, and the entire plot was surrounded by wire fencing to prevent stray wild dogs (*Canis lupus familiaris*) or Tibetan foxes (*Vulpes ferrilata*) from destroying the yak fur (**Supplementary Figure S2**).

On the morning of November 23, 2022, the above seven seed species were manually adhered to yak fur using a tweezer (i.e., simulating seed adhesion to yak flank) (**Supplementary Figure S2**). *Salvia* seeds soaked in water before the adhesion simulation. There were 30 seeds per signboard and three signboard replicates for each species. There were three blank signboards (i.e., controls) to detect any seeds that may have come into contact with the yak fur as a result of wind or other factors. No seeds were found on the control group yak fur during the entire experimental period. The number of seeds retained on each signboard was recorded at 12-h intervals. After each count was completed, the positions of two adjacent signboards were switched (simulating livestock movement). The direction of each signboard also random changed throughout the experiment. The duration of the simulated adhesion test was 252 h (10.5 days). Then, we calculated the percentage of seeds retained at each time point for each signboard. In addition, a regression analysis was carried out with Origin 2021 software to construct the relationship between seed retention rate, adhesive structure length, and retention time.

### 2.3 Observation of yak grazing behavior

We observed the yaks from a single herding family to determine grazing behavior. These yaks (200 head, 80% of which were 3.5- to 5-year-old females, with an average shoulder height of ~1.2 m and weight of ~250 kg) were the only grazing domestic livestock on this family’s seasonal grazing pastures, except for a very small number of free-range horses.

Yak grazing behaviors were observed in their seasonal grazing pastures during early January (winter), late April (i.e., spring), mid-July (summer), and late October (autumn) of 2022, with three consecutive days of observation during each season (**Supplementary Figure S5**). The four seasonal pastures are >30 km apart from each other, and the area of each seasonal pasture is >100 ha. During the observation period, three adult female yaks were observed with binoculars at 1-hour intervals from the beginning of grazing in the morning (at which point they have been herded from the night pen to the pasture) to the end of grazing (when they return to the night pen) during a whole grazing day, and each yak was observed continuously for 10 minutes during each 1-hour interval.

During our observation period, we made note of both primary and secondary behavioral patterns. Primary grazing behavior refers to the temporal distribution of such behaviors that generally include moving and stationary/motionless states. Briefly, moving refers to cross walking on the front legs and hindquarters of the yak, which includes foraging (i.e., when yaks kept their heads close to the ground and were biting or searching for forage, including movements such as biting, chewing, ingestion, and head-shaking) and walking (described as a continuous activity, during which animals showed no sign of grazing and walked with their heads up), therefore, foraging and walking do not occur at the same speed. We note that yaks that did not move their hind legs and instead simply rotated their body on the axis of the hind legs to forage were not included. A stationary behavior includes ruminating/resting (i.e., when animals were in a standing or lying position and includes activities such as chewing, swallowing, and regurgitation of boluses of ingesta), drinking, excretion (of dung and/or urine), grooming, wallowing, and peer interactions, etc. as those activities are time consuming (Hilario et al., 2017; Yang et al., 2021). Secondary behavioral patterns mainly included the number of steps per minute and the distance of each step (using track-related/hoof-print measurements). Observations were made at a distance of ≥50 m away from the herd to avoid interfering with the normal activities of the yaks.

### 2.4 Yak epizoochory seed dispersal distance estimation

We first calculated the seed retention time on yak fur on the basis of the regression results from the seed adhesion test. The relationship between seed retention rate, retention time, and adhesive structure length was described by the equation *Z* = *aX* + *bY* + *c*, where *Z* represents the seed retention rate, *X* is the retention time, *Y* is the adhesive structure length, *a* and *b* are the regression coefficients, and *c* is the intercept; and the value of *X* was obtained when *Z* = 0.

We then calculated the yak epizoochorous seed dispersal distance using the following equations (Equations 1–4):


(1) $$\text{Seed straight line dispersal distance}\approx \frac{\text{retention time}}{\text{walking time}}\times \text{walking distance per day}$$

(2) Walking distance per day=distance per step (m)×walking steps per day

(3) Walking steps per day=foraging rate (steps/min)×walking time per day (min)

(4) Grazing time=walking time+resting time+standing time…

For *S. roborowskii* seeds, as the adhesive structure is mucilage, it cannot be measured with a length unit and is theoretically infinite. Therefore, the epizoochorous dispersal distance of *S. roborowskii* seeds should be greater than the maximum distance of the other six species.

It should be noted that although yaks are semi-feral animals, for safety reasons and to avoid predation by wolves or wild dogs, the herders keep these animals during certain seasons in a night pen (i.e., at their campsites) and then move them to grazing areas (i.e., pastures) during the day. They are then returned to the night pen (campsites) at night. In this case, the location of the campsites is not fixed, but they are set up close to the location where the herd is grazing. Therefore, the epizoochory dispersal distance in this study is an estimate of the shortest straight-line distance traveled by yaks during the retention time. However, when seasonal long-distance migration events of herds occur, this estimated value should approximate the value of the actual direction-specific distance traveled by the herd.

### 2.5 Statistical analysis

A one-way analysis of variance (ANOVA) was used to compare the variability in primary grazing behavior (i.e., foraging time) and secondary grazing behavior (foraging rate and per step distance) of yaks between seasons, with season set as a fixed factor. In addition, a two-way ANOVA was used to compare the variability in the dispersal distance of seeds from seven plant species between seasons, with season and seed species (length of adhesive structures) set as fixed factors. The ANOVAs were all carried out with SPSS 27.0 software, and multiple comparisons were performed using Duncan’s method with a significance level of *P*< 0.05.

## 3 Results

### 3.1 Retention rate of seeds adhered to yak fur

**Figure 1:**
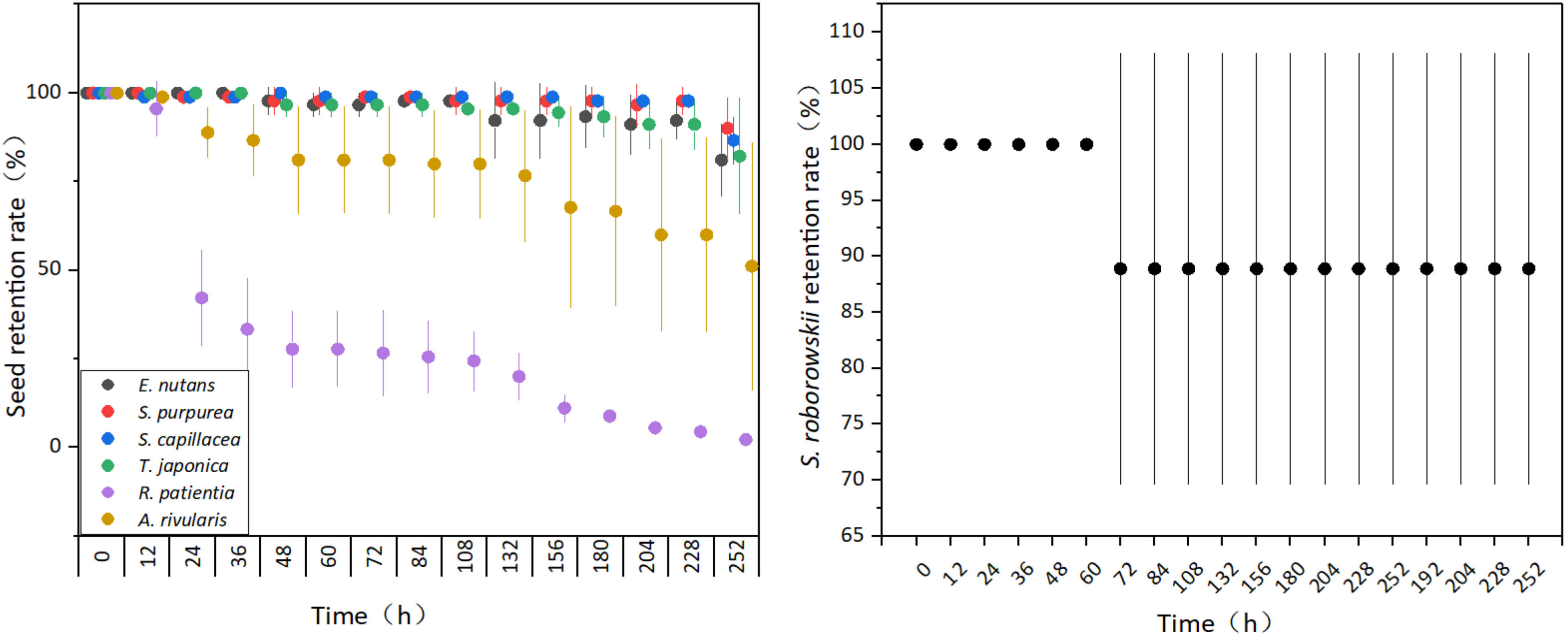
Temporal dynamics of seed retention rates on yak fur in an experimental system. Error bars indicate standard error (SE), for each specie, three signboards were analyzed for each time point (**Supplementary Figure S2**).

The retention rates of the seven species of seeds that were experimentally adhered to yak fur all gradually decreased over time. After 12 h, the retention rates of seeds from *S. capillacea* (98.89%), *R. patientia* (95.56%), and *A. rivularis* (98.89%) began to decrease; after 24 h, the *S. purpurea* rate had also decreased (98.89%); and after 48 h, the rate of *E. nutans* (97.78%) and *A. coelestina* (96.67%) had decreased. After 252 h, the lowest seed retention rate was 2.22% for *R. patientia*, and the highest was 86.67% for *S. capillacea* (**Figure 1**).

The seed retention rate of *S. roborowskii* decreased after 72 h of adherence but then remained at 88.89% until the end of the observation period (252 h; **Figure 1**).

### 3.2 Correlation between seed retention rate, time and adhesive structure length

**Figure 2:**
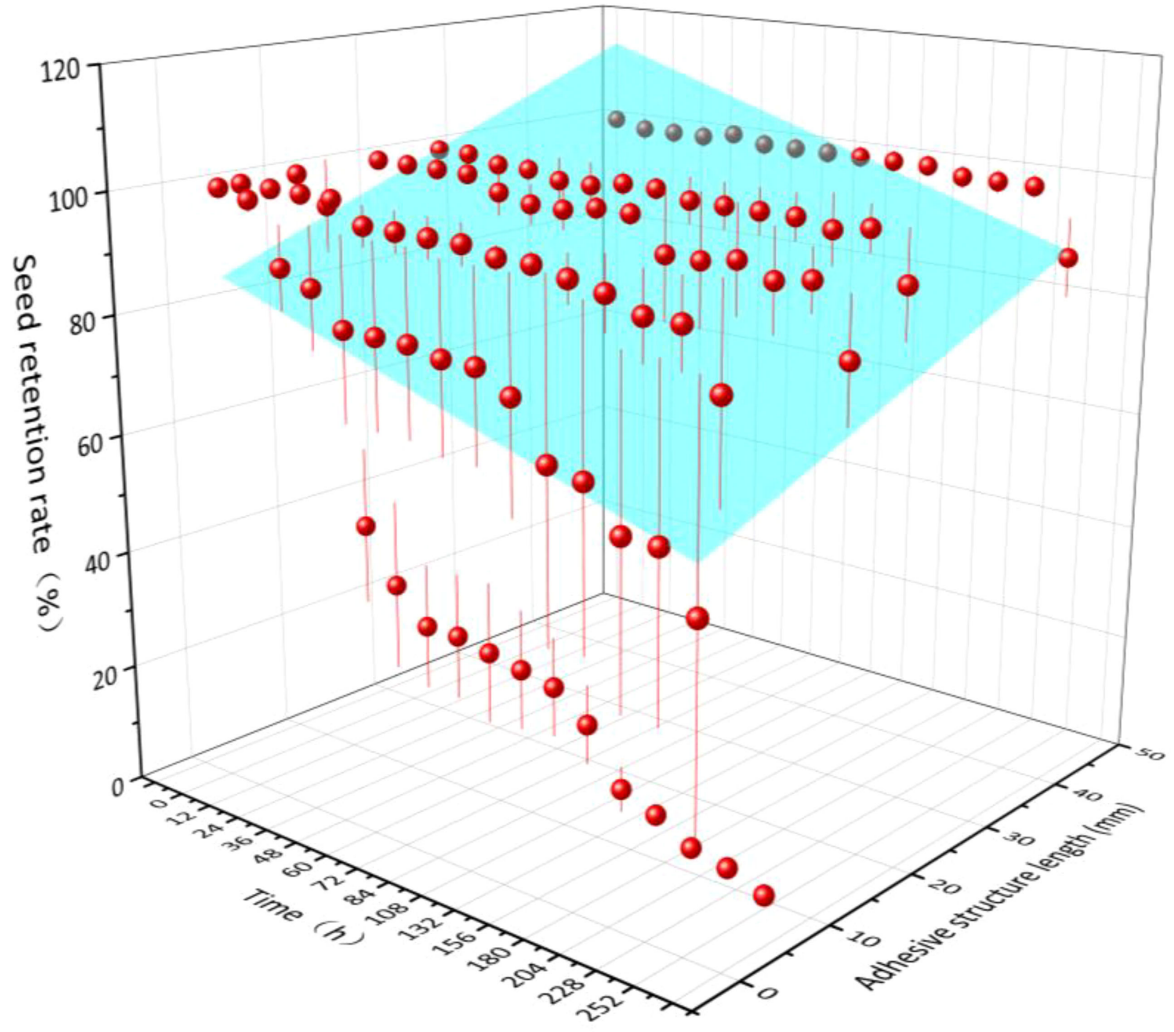
Relationship between seed retention rate and retention time, as well as the length of adhesive structures for the six seed species with such structures examined here.

Over time (*X*), seed retention rate (*Z*) increased with increasing length of adhesive structures (*Y*), and the relationship among those three indexes conformed to the equation *Z* = –1.89*X* + 0.64*Y* + 86.63 (*R* ^2^ = 0.19, *P*< 0.01). That is, the seed retention rate was significantly negatively correlated with adhesive time and positively correlated with adhesive structure length (**Figure 2**).

### 3.3 Yak behavioral patterns across seasons

**Figure 3:**
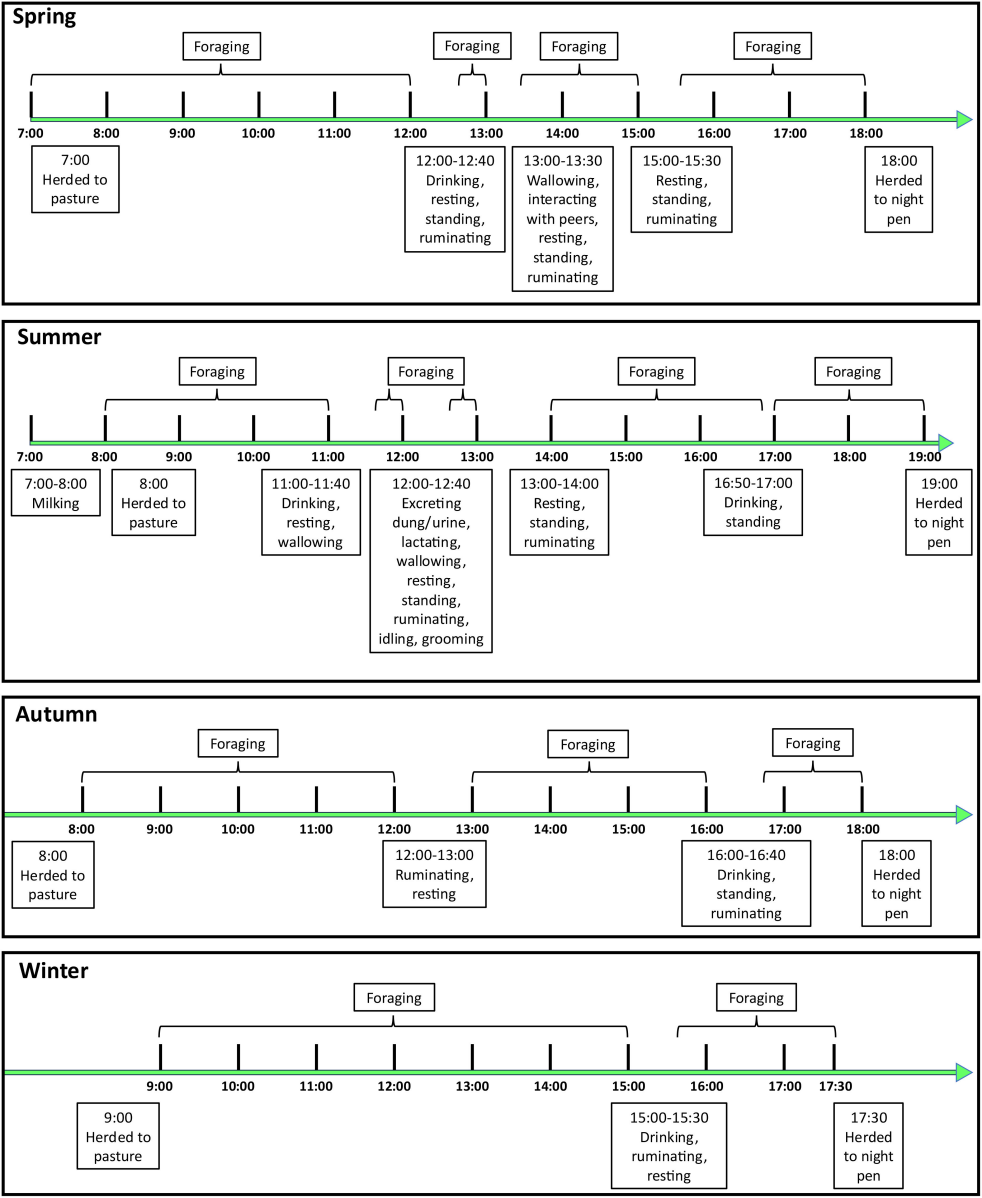
Behavioral patterns of grazing yaks across the seasons. Three yaks were observed during each day and three consecutive days were observed during each season.

The yak foraging/walking time during the day was 560 min during the spring, which is significantly higher than the other three grazing seasons (*P*< 0.05). Foraging/walking time during the summer (510 min) and autumn (500 min) were not significantly different (*P* > 0.05); however, they were significantly higher than foraging/walking time during the winter (480 min) (**Figure 3**).

### 3.4 Yak foraging rate dynamics across seasons

**Figure 4:**
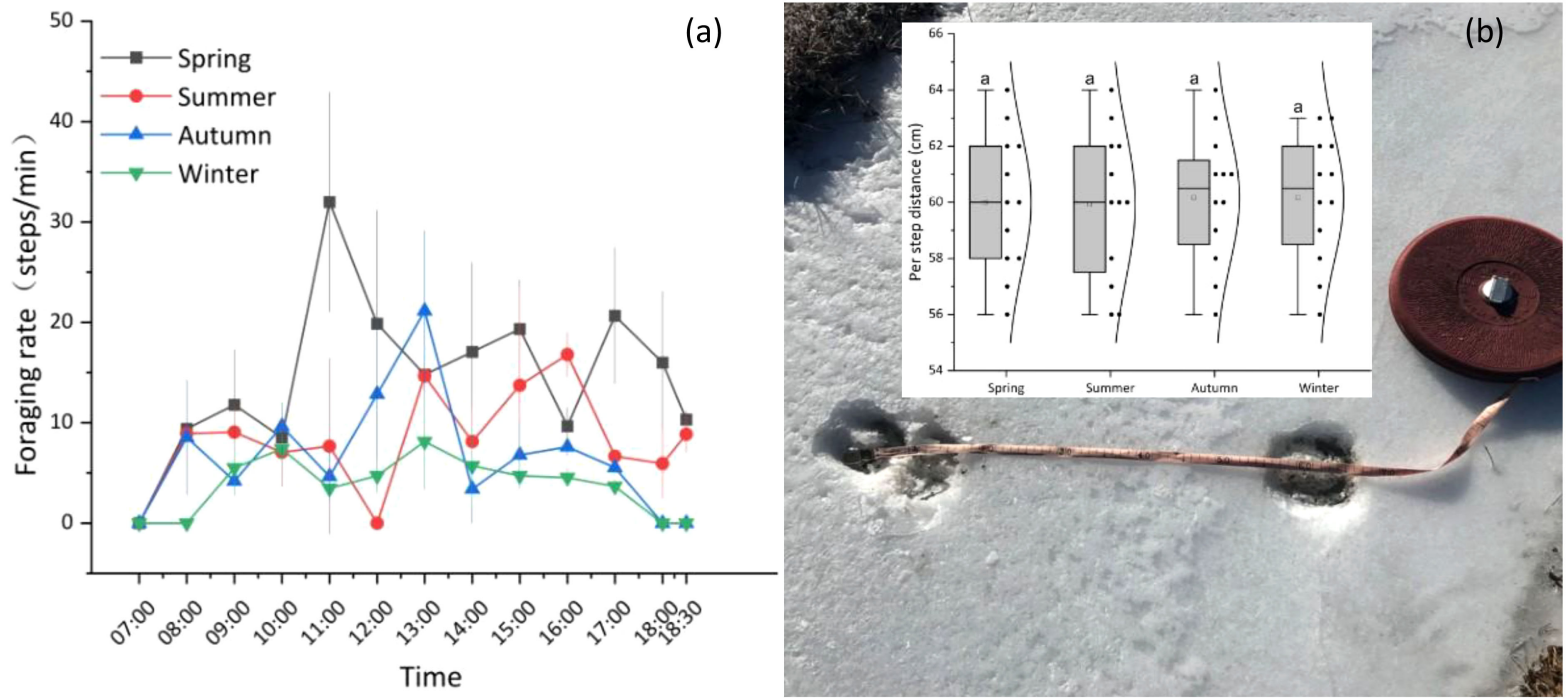
Seasonal dynamics of yak foraging rates **(a)** and distance traveled per step **(b)**. **(a)** Error bars indicate standard error (SE); n = 9 yaks per season; **(b)** Track-related/hoof-print measurements were used to determine per step distance, 9 yaks were used for making measurements and 12 hoof-prints were measured per season, there were no significant differences across seasons, as indicated by same lowercase.

The average foraging rate during the spring (15.79 steps/min) was significantly higher than that of the other three seasons (*P*< 0.05), whereas the foraging rates during the summer (8.96 steps/min) and autumn (8.44 steps/min) were not significantly different (*P* > 0.05). In contrast, these rates were significantly higher than the winter foraging rate (5.32 steps/min; *P*< 0.05). Overall, the yak foraging rate across all seasons is characterized by an ‘M’-shaped bimodal curve, with one foraging peak in the morning (9:00–11:00) and the other in the afternoon (15:00–17:00), with a trough corresponding to the mid-afternoon period (12:00–14:00) (**Figure 4a**). Grazing yaks had an average per step distance of 60 cm (**Figure 4b**).

### 3.5 Yak epizoochorous seed dispersal distances across the seasons

**Figure 5:**
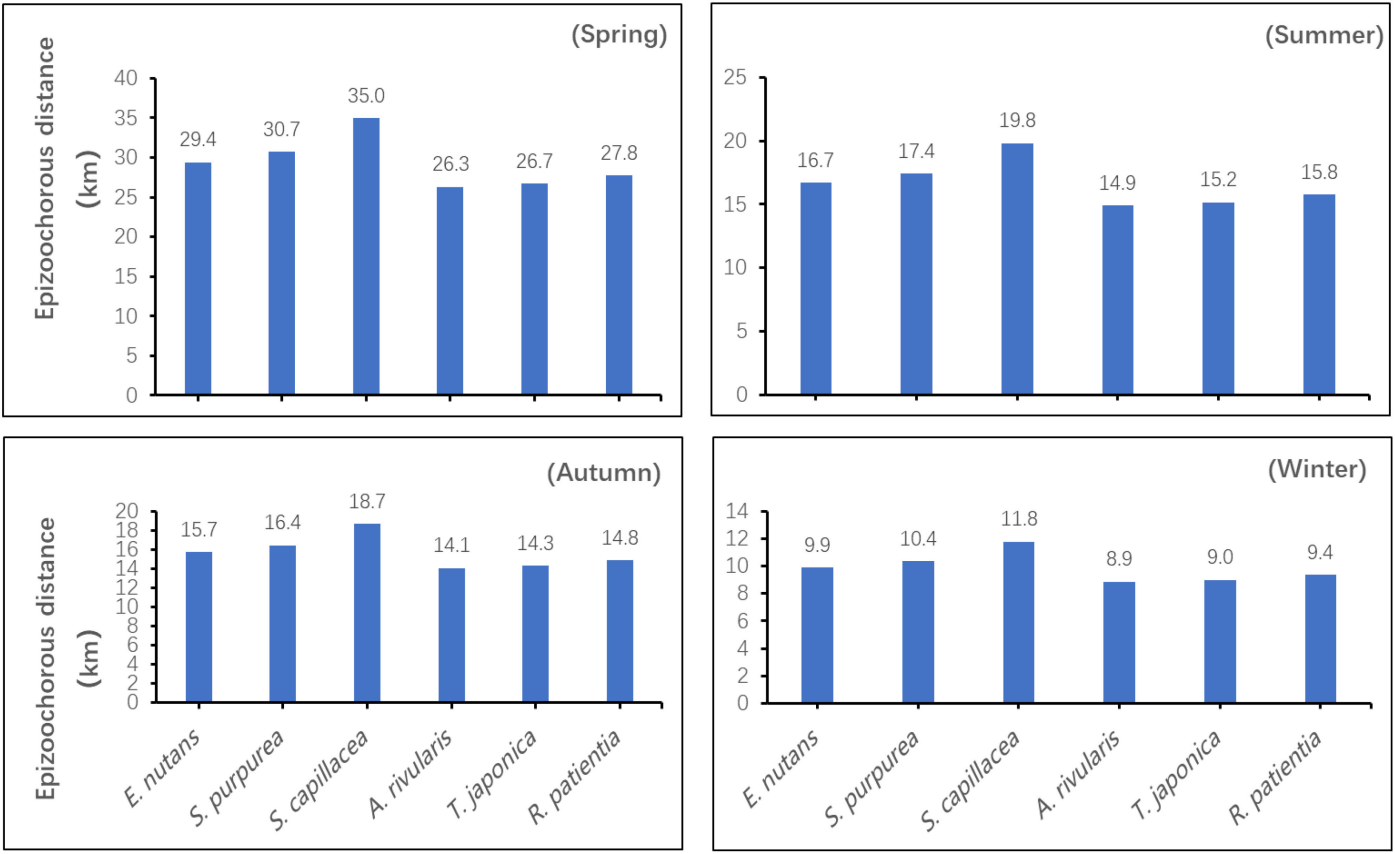
Estimated epizoochorous seed dispersal distances for six plant species by grazing yaks across seasons.

Two-way ANOVA showed that, the season and seed species had significant effects on the epizoochorous dispersal distance, and there was a significant interaction effect as well (*P*< 0.05). For a given seed species, the epizoochorous dispersal distances were ranked as follows: spring > summer > autumn > winter. During a single season, seeds with longer adhesive structures had greater epizoochorous dispersal distances. During the spring, *S. capillacea* seeds had the greatest dispersal distance, which was 34.95 km. During the winter, *A. rivularis* seeds had the shortest dispersal distance, which was 8.86 km (**Figure 5**).

## 4 Discussion

### 4.1 Relationship between seed adhesive structures and retention time and retention rate on animal fur

In our study, appendage structure specialization facilitated epizoochory seed dispersal, and the length of the adhesive structure was significantly positively correlated with retention time and retention rate on the yak fur (**Figure 2**; Wang et al., 2021), thus making the seeds more conducive to long-distance dispersal. The retention rate of the three Poaceae species seeds, all of which have awns, was >80% after 252 h of adhering to yak fur (**Figure 1**). Another member of the Poaceae family, *Hordeum murinum*, which also has awns, has seeds with a 9.6% retention rate after 28 days of adherence to sheep fur (Manzano and Malo, 2006). The mucilaginous seeds in this study—*S. roborowskii*—had an unchanged retention rate from 72 h to 204 h after adherence (**Figure 1**); their unique type of appendage structure resulted in a dispersal distance that could not be measured in terms of a specific amount as their adhesive structure length is theoretically infinite.

Appendage structures are advantageous for epizoochory dispersal (Graae, 2002; Albert et al., 2015). However, seeds with non-specialized adhesive structures can still adhere to animal fur and undergo epizoochory (Fischer et al., 1996; Couvreur et al., 2004; Anders et al., 2009; Wang et al., 2021), in which cases the influence of animal fur characteristics is significant. A simulated adhesion test for seeds indicates that thick furs with long, rough, undulating hairs implanted at a large angle are most suited for seed adhesion, and this adhesion occurs independently of whether the seeds have adhesive structures or not (Couvreur et al., 2004). In this study, yaks deep furs with long, rough, undulated hairs implanted at a large angle, which are conducive for seed adhesion (**Supplementary Figure S4**). It has even been suggested that epizoochory is a relatively rare dispersal system for plants as a whole, as the percentage of plant species with seeds adapted for transport on the outside of animals is estimated to be<5% (Sorensen, 1986; Iluz, 2011). Thus, seed adhesion time, as well as the retention rate on animal fur, is the result of a combination of both the seed morphology and the characteristics of the animal’s fur.

In addition, the position of the seed on the animal’s body surface also has an important impact on the retention rate as well as retention time. For example, seed retention rate on the back of the animal is higher than that on the flank (Kiviniemi, 1996; Graae, 2002). Adding another layer of complexity, seeds with obvious appendage structures may have a greater chance of detection by animals, which may cause behaviors such as grooming or shaking of the body, thus promoting seed dropping (Kiviniemi, 1996; Manzano and Malo, 2006). Otherwise, seeds that are not detected by the animal may be transported until they drop or rot or the animal sheds its coat (Sorensen, 1986). Therefore, there exists a trade-off, whereby pronounced adhesive structures on seeds promote their attachment to fur but may reduce the probability of successful dispersal by increasing grooming behavior, especially if the seeds are accessible and noticeable by the animals (Kiviniemi, 1996).

### 4.2 Seasonal variations of yak grazing behaviors

Seed adhesion to animal fur is a prerequisite for epizoochory dispersal. The behaviors of those animals, which act as carriers with autonomous mobility, especially with respect to their walking time and activity range, determine the final dispersal distance of the attached seeds. For large herbivores, grazing behaviors are influenced by factors such as the environment, weather, temperature, geographical conditions, sward surface height, time on pasture, age, herbage allowance, stocking density and botanical composition of pastures (Hilario et al., 2017). In this study, both primary (e.g., walking time) and secondary (e.g., foraging rate) behavioral patterns of yaks showed an obvious seasonal dynamic (**Figures 3**, **4**). During the spring, as the temperature rises and the herbage begins to green up, however, the yaks in this environment are at their lowest level of physical fitness after surviving a severe winter. They must regain their physical condition by foraging quickly, which is the reason why the foraging time and rate increase significantly during this period. Liu et al. (2014) also found that the proportion of foraging time for yaks during the spring (34.18%) is higher than that during other seasons in the Helan Mountain region of northwest China. During the warm season (i.e., summer and autumn), the aboveground biomass and nutrient quality of forage are at their peak. As the yaks do not have to walk as far or as quickly to access adequate herbage, more time can be allocated to other behaviors (e.g., resting, ruminating etc.) (**Figure 3**; Liu et al., 2019). During the winter, when the temperature decreases (less than –15°C) and the herbage consists of standing litter with low nutritional quality, the yaks save energy by reducing their foraging/walking rate, although they allocate more time to foraging to obtain enough food to survive the harsh winter (the yaks in this study received no supplemental feeding). In addition, as the period of daylight is shortened in the winter, the yaks are herded to the pasture later and return to the night pen early; thus the grazing time is reduced, which also leads to a reduction in foraging time during this season. Liu et al. (2019) found that both meteorological and forage conditions affected the grazing behavior of yaks and that the general reduction in grazing activities in winter could have been a strategy to reduce exposure to the harsh conditions and, consequently, to reduce energy expenditures for foraging and for thermoregulation.

### 4.3 Epizoochory dispersal distances of plants seeds

As compared with other dissemination mechanisms (e.g., hydrochory, anemochory, ballochory, etc.), epizoochory can cover greater distances and may also be directed as a result of management practices (e.g., for livestock), the animals’ own behavioral traits, and their activity patterns (Manzano and Malo, 2006; Albert et al., 2015). For example, under traditional transhumance in Spain, Merino sheep could spread seeds up to 400 km away on the basis of fur adhesion, even across several plateaus and valleys as they make their progress (Manzano and Malo, 2006). In this study, the farthest epizoochory dispersal distance for alpine meadow plant seeds by yaks under seasonal grazing management practices was estimated to be ~35 km (**Figure 5**).

In grassland ecosystems, large herbivores play an important role in the epizoochory dispersal of herbage seeds. In a coastal dune nature reserve in Belgium, 13 plant species are dispersed by epizoochory by free-ranging donkeys, with a range of transmission of ~100 ha; they account for 20% of the number of local plant species (Couvreur et al., 2005). In the pastures of southern Sweden, *Agrimonia eupatoria* L., *Geum rivale* L. and *Triglochin palustre* L., all of which have adhesive structures on their seeds, may be dispersed from tens of meters to a kilometer by fallow deer (*Dama dama*) and domestic cattle (*Bos taurus*) (Kiviniemi, 1996). In the calcareous grasslands of southwest Germany, the results of a seed adhesion test of sheep epizoochory showed that ~8% of adhesive caryopses were retained for >7 months, during which the flock had covered a distance of well over 100 km (Fischer et al., 1996). It is worth noting that for migratory wild herbivores with a much wider range, such as Mongolian gazelle (*Procapra gutturosa*), with a migration distance of >18000 km (Nandintsetseg et al., 2022), epizoochory-based dispersal could, theoretically involve even greater distances, yet not much research has been carried out on this topic due to the challenge of the experimental methods. Otherwise, because numerous wild ungulates inhabit alpine regions and because their seasonal migrations may cover a vast territory, the place of wild herbivores in the seed-dispersal process (both on their body and through ingestion and deposition) needs further exploration. In short, grazing livestock transport grassland plant seeds through epizoochory and thus promote grassland regeneration, development and biological conservation, which explains the significance of grazing as a suitable management practice for maintaining grassland health.

### 4.4 Limitation and prospect of the study

It should be noted that, in this study, a static seed adhesion simulation experiment was used to assess the epizoochory dispersal distance in an alpine meadow, however, the reality is that epizoochorous seed dispersal is dynamic, and the grazing behaviors (e.g., grooming, wallowing and running, etc.) of livestock may have an positive effect on seed detachment (Kiviniemi, 1996), yet the static simulation experiment is unable to reflect this point, which should be a shortcoming of this study. Moreover, the movement trajectory of yaks may not always follow a straight line, whereas the application of GPS technology could map the movement trajectory of herds (Nandintsetseg et al., 2022), which should help to solve this problem, and this also provide an idea for future research direction.

Intensified anthropic activities have substantially degraded grasslands in the alpine region of the Qinghai-Tibetan Plateau, resulting in greatly fragmented habitats and heterogeneity among grassland areas. By combining a simulated seed adhesion test with data on the grazing behavior of large herbivores, this study demonstrates that large herbivores have the capacity to connect grassland patches scattered in different regions through epizoochory seed dispersal, thus promoting the integration of different grassland communities. Because numerous wild ungulates inhabit alpine regions and because their seasonal migrations may cover a vast territory, the place of wild herbivores in the seed-dispersal process (both on their body and through ingestion and deposition) needs further exploration.

## 5 Conclusion

Large herbivores (e.g., yaks) in alpine regions are important vectors for long-distance dispersal of herbage seeds, both through epizoochory and endozoochory (see Wang and Hou, 2021; Wang and Hou, 2025), and these two modes of dispersal are complementary. Under seasonal grazing management, the transport of domestic livestock promotes the blending of plant communities (e.g., gene flow, plant community assembly) between different rangelands, especially in areas with fragmented habitats. These livestock act as ‘ecology corridors’ and thus can connect isolated patches of grassland (Albert et al., 2015). Therefore, we emphasize that seasonal grazing is an appropriate management practice as well as conservation strategies for coping with the highly fragmented status of grassland habitats in the alpine region.

## Data Availability

The datasets presented in this study can be found in online repositories. The names of the repository/repositories and accession number(s) can be found below: The relevant experimental data are available from Figshare and can be accessed through the following link: https://doi.org/10.6084/m9.figshare.25752015.
