# Supplementary material for: Estimated epizoochory seed dispersal distances by grazing yak across seasons in an alpine meadow
Source: Front Plant Sci. 2025 Jun 12;16:1569043. doi: 10.3389/fpls.2025.1569043 (PMC12197954; doi:10.3389/fpls.2025.1569043)
Supplement: Supplementary file 1 [file DataSheet1.docx]

**Supplementary Material**

Supplementary Material for: Estimated epizoochory seed dispersal distances by grazing yak across seasons in an alpine meadow

**This file includes:**

**Figures:** FIGURE S1 - S5

**Table:** Table S1

**1. Vegetation type profile of the study area**

The four seasonal pastures are all alpine meadows with a complex community structure and high species diversity (~30 species/m^2^). The constructive species are Cyperaceae plants (e.g., *Carex hughii* S. R. Zhang, *Carex parvula* O. Yano and *Carex thibetica* Franch.). Dominant species are Compositae plants (e.g., *Saussurea hieracioides* Hook. f., *Anaphalis lactea* Maxim. and *Aster diplostephioides* (DC.) C. B. Clarke), Ranunculaceae plants (e.g., *Anemone rivularis* var. *flore-minore* Maxim., *Anemone coelestina* var. *linearis* (Bruhl) Ziman and B. E. Dutton and *Thalictrum alpinum* L.) and Gentianaceae plants (e.g., *Gentiana lawrencei* var. *farreri* (I. B. Balfour) T. N. Ho, *Gentianopsis paludosa* (Hook. f.) Ma and *Halenia elliptica* D. Don). Additionally, there are scattered shrubs *Hippophae tibetana* Schlechtendal and *Dasiphora fruticosa* (L.) Rydb. The soil type is alpine-meadow soil with soil layer thickness of 50–70 cm, with >80% of the belowground biomass being concentrated in the soil at a depth of 0–20 cm. The study area has undulating mountains of steep, variable, complex and fragmented terrain.

**2. Climate type**

The site for this study has a plateau-type climate with cold temperatures and relatively high humidity. There is only a warm season (early May to late November) and cold season (early December to late April), with absolutely no frost-free period during the year. The period during which frost occurs consists of ~270 days annually. According to meteorological data provided by local weather stations, the average annual temperature for the study area over the past 40 years is ~1.2°C, with the highest average temperature (11.9°C) occurring in July and the lowest average temperature (–8.9°C) in January. The average annual precipitation is ~620 mm, mainly concentrated during the forage growing season (May to August) (Figure S1).


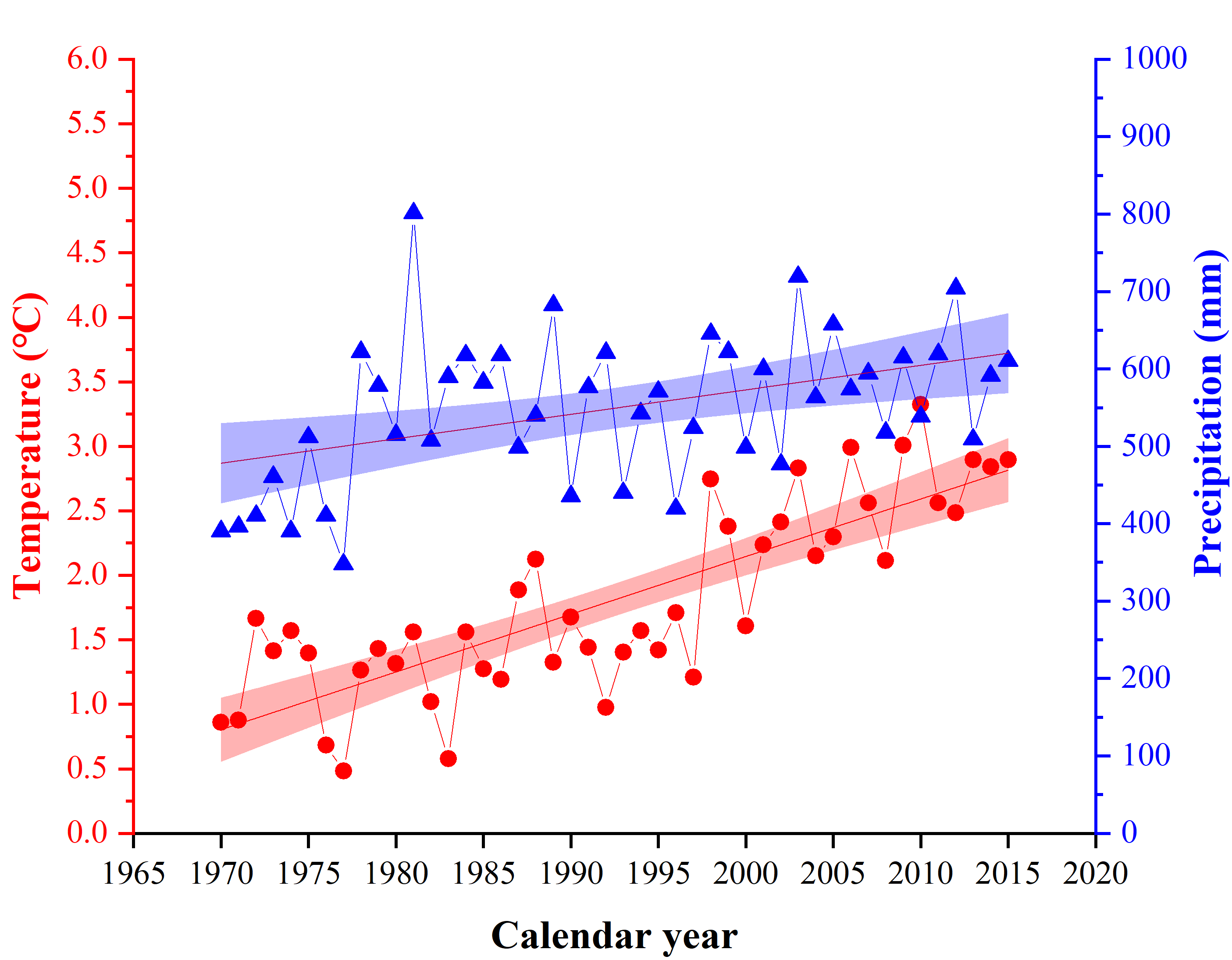


**FIGURE S1** Interannual variation and monotonic trends in annual temperature (red) and precipitation (blue) at the Maqu Research Station, Northeast Qinghai-Tibetan Plateau. Lines denote linear fit; shaded areas around the regression lines denote 95% confidence intervals. Temperature: *y* = 0.0447*x* – 87.335 (*R*^2^ = 0.672, *P* = 0.000); precipitation: *y* = 3.160*x* – 5747.062 (*R*^2^ = 0.190, *P* = 0.00245). Meteorological data were provided by the Maqu Research Station.


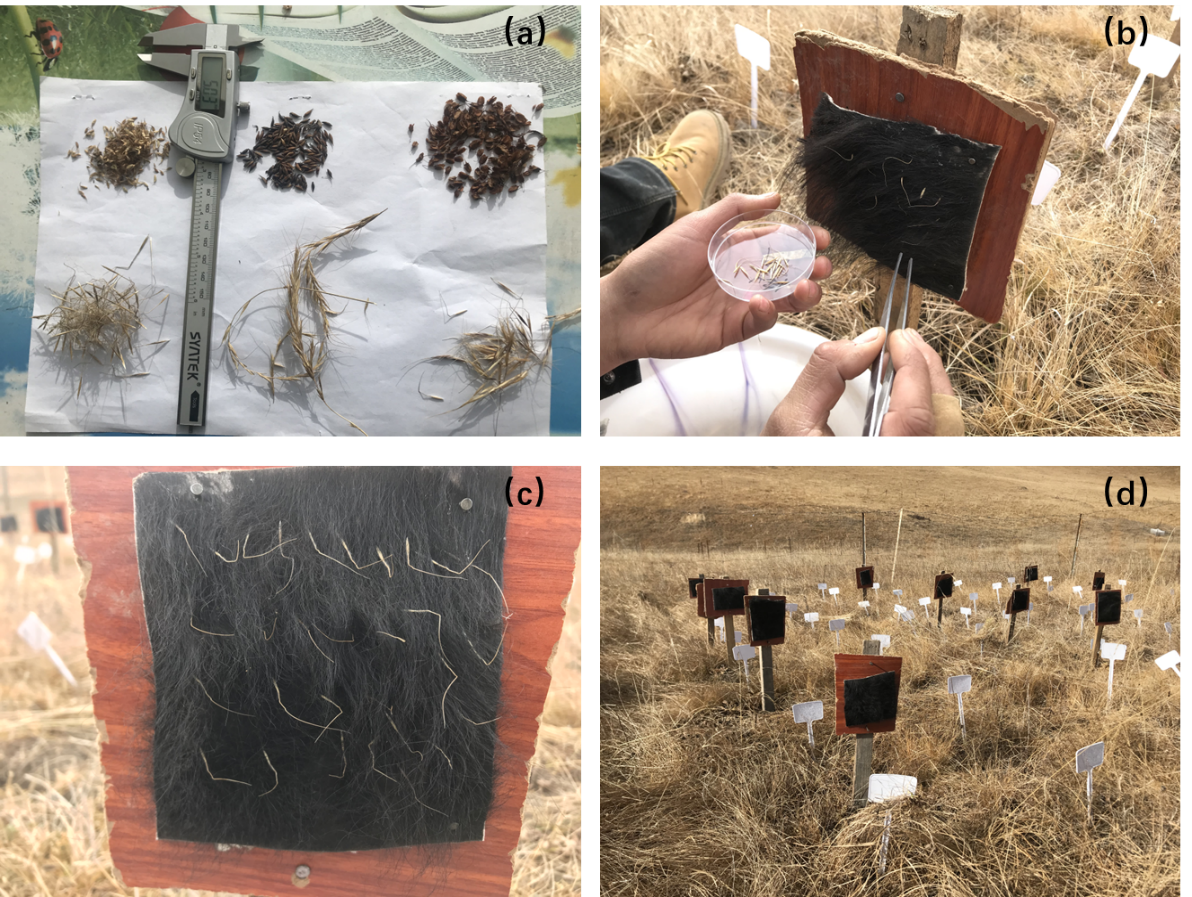


**FIGURE S2** Simulation test of attachment of six alpine meadow plant species seeds (except for *Salvia* seeds, see below Figure S3) to yak fur. (a) Six species seeds; (b) Seed adhesion simulation test; (c) Seed attached on yak fur and (d) the signboards were staked in the fenced natural grassland.


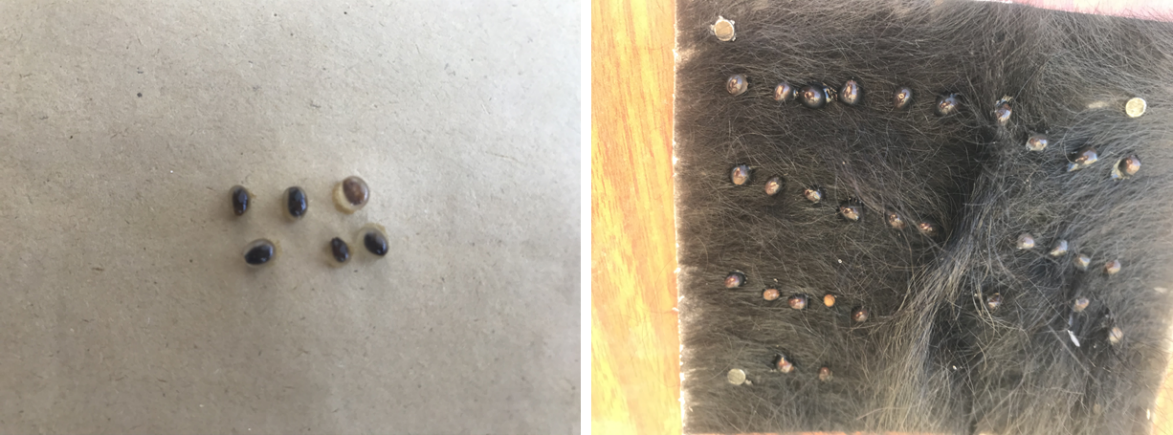


**FIGURE S3** Simulation test of attachment of *Salvia roborowskii* Maxim. seeds to yak fur. Moisture was introduced to the yak fur sample.


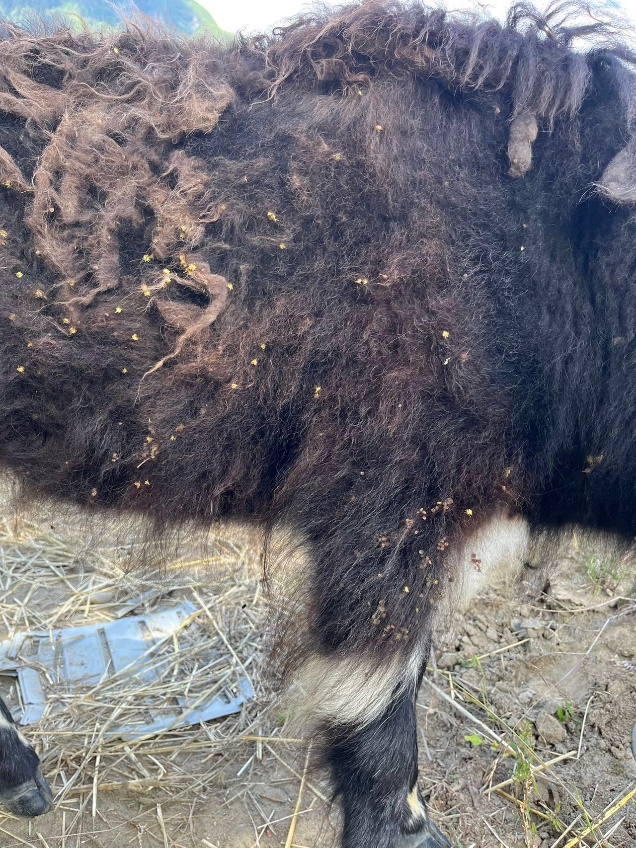


**FIGURE S4** Seed attached to yak fur. This image represents the natural occurrence of seed attachment.


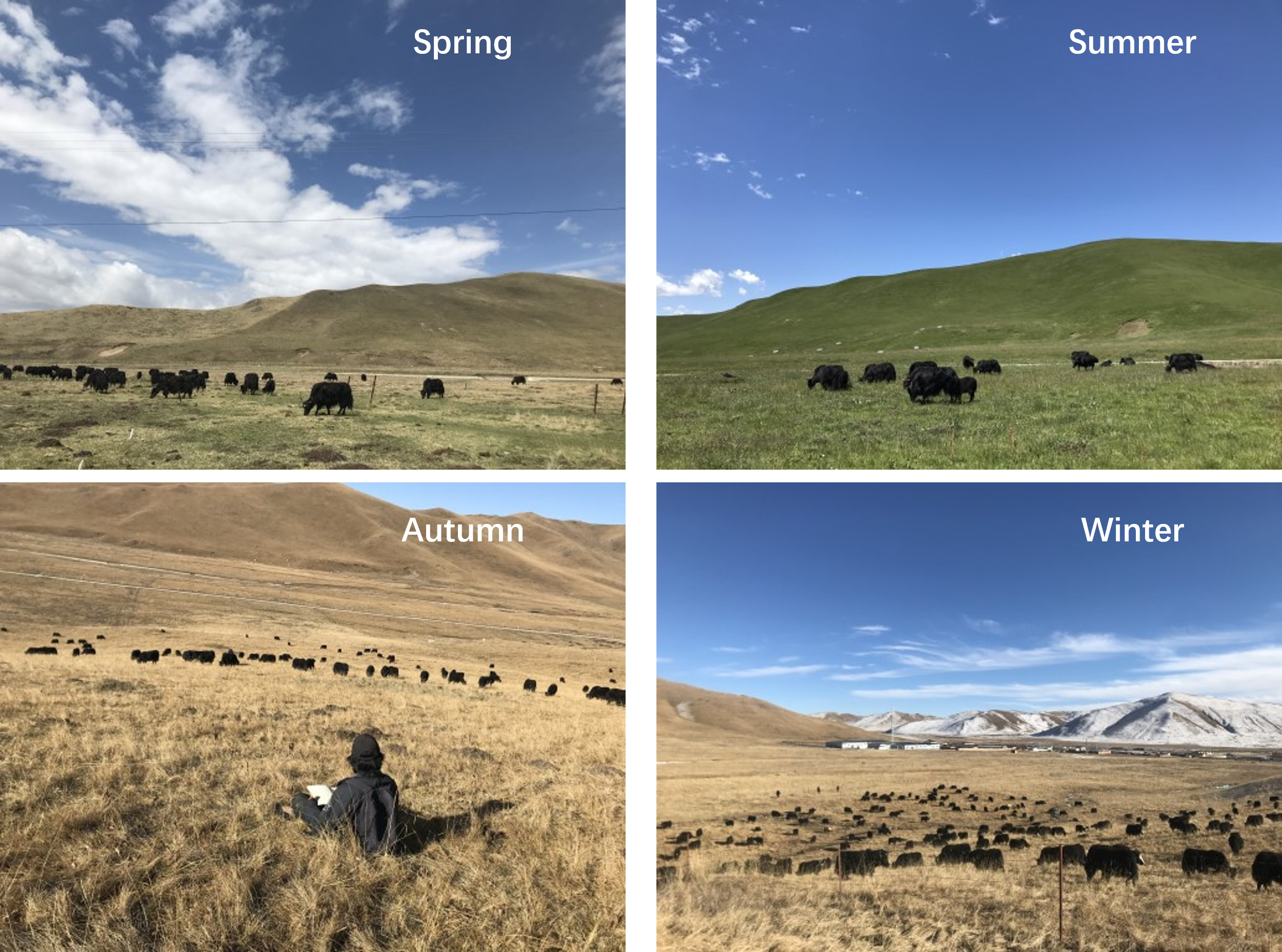


**FIGURE S5** Observation of yak grazing behaviors.

**Table S1** Adhesive structure type and length of seeds from seven plant species

| Family | Species | Reproductive branches height (cm) | Adhesive structure | Length (mm) ^a^ |
| --- | --- | --- | --- | --- |
| Poaceae | *Elymus nutans* Griseb. | 100 | Awn | 17.49 ± 1.85 |
|  | *Stipa purpurea* Griseb. | 50 | Awn | 24.29 ± 2.17 |
|  | *Stipa capillacea* Keng | 50 | Awn | 46.23 ± 9.49 |
| Ranunculaceae | *Anemone rivularis* var. *flore-minore* Maxim. | 65 | Hook | 1.28 ± 0.22 |
|  | *Anemone coelestina* var. *linearis* (Brühl) Ziman & B. E. Dutton | 20 | Bristle, Hook | 3.46 ± 0.36 |
| Polygonaceae | *Rumex patientia* L. | 150 | Wings, Hook | 8.96 ± 2.03 |
| Lamiaceae | *Salvia roborowskii* Maxim. | 90 | Mucilage | -- |

Note: -- indicates that the shape of this seed was not suitable for length measurements. ^a^ The average length of the adhesive structures was determined from 12 seeds and is shown ± the standard error.
